# Trends in the incidence of chronic fatigue syndrome and fibromyalgia in the UK, 2001–2013: a Clinical Practice Research Datalink study

**DOI:** 10.1177/0141076817702530

**Published:** 2017-03-30

**Authors:** Simon M Collin, Inger J Bakken, Irwin Nazareth, Esther Crawley, Peter D White

**Affiliations:** 1 School of Social and Community Medicine, University of Bristol, Bristol BS8 2BN, UK; 2 Norwegian Institute of Public Health, 0403 Oslo, Norway; 3 UCL Department of Primary Care and Population Health, UCL Royal Free Campus, London NW3 2PF, UK; 4 Wolfson Institute of Preventive Medicine, Barts and the London School of Medicine and Dentistry, Queen Mary University of London, London EC1M 6BQ, UK

**Keywords:** Chronic fatigue syndrome, myalgic encephalomyelitis, fibromyalgia, post-viral fatigue, primary care, general practice, diagnosis, incidence; fatigue

## Abstract

**Objective:**

Trends in recorded diagnoses of chronic fatigue syndrome (CFS, also known as ‘myalgic encephalomyelitis’ (ME)) and fibromyalgia (FM) in the UK were last reported more than ten years ago, for the period 1990–2001. Our aim was to analyse trends in incident diagnoses of CFS/ME and FM for the period 2001–2013, and to investigate whether incidence might vary by index of multiple deprivation (IMD) score.

**Design:**

Electronic health records cohort study.

**Setting:**

NHS primary care practices in the UK.

**Participants:**

Participants: Patients registered with general practices linked to the Clinical Practice Research Datalink (CPRD) primary care database from January 2001 to December 2013.

**Main outcome measure:**

Incidence of CFS/ME, FM, post-viral fatigue syndrome (PVFS), and asthenia/debility.

**Results:**

The overall annual incidence of recorded cases of CFS/ME was 14.8 (95% CI 14.5, 15.1) per 100,000 people. Overall annual incidence per 100,000 people for FM was 33.3 (32.8–33.8), for PVFS 12.2 (11.9, 12.5), and for asthenia/debility 7.0 (6.8, 7.2). Annual incidence rates for CFS/ME diagnoses decreased from 17.5 (16.1, 18.9) in 2001 to 12.6 (11.5, 13.8) in 2013 (annual percent change −2.8% (−3.6%, −2.0%)). Annual incidence rates for FM diagnoses decreased from 32.3 (30.4, 34.3) to 27.1 (25.5, 28.6) in 2007, then increased to 38.2 (36.3, 40.1) per 100,000 people in 2013. Overall annual incidence of recorded fatigue symptoms was 2246 (2242, 2250) per 100,000 people. Compared with the least deprived IMD quintile, incidence of CFS/ME in the most deprived quintile was 39% lower (incidence rate ratio (IRR) 0.61 (0.50, 0.75)), whereas rates of FM were 40% higher (IRR 1.40 (0.95, 2.06)).

**Conclusion:**

These analyses suggest a gradual decline in recorded diagnoses of CFS/ME since 2001, and an increase in diagnoses of fibromyalgia, with opposing socioeconomic patterns of lower rates of CFS/ME diagnoses in the poorest areas compared with higher rates of FM diagnoses.

## Introduction

Chronic fatigue syndrome (CFS, also known as myalgic encephalomyelitis (ME)) and fibromyalgia (FM) are chronic diseases that share superficial similarities, including unknown aetiology and pathophysiology, varied symptomatology, a wide range of severity, higher incidence in women, no laboratory test to confirm diagnosis (only to rule out other diagnoses), and specific co-morbidities.^1,2^ Three-quarters of FM patients report being fatigued,^3^ and one-fifth of adult and paediatric CFS/ME patients report widespread pain.^4^ Both diseases are debilitating, typically imposing substantial burdens on patients, carers, and families.^5,6^

Seven case definitions for CFS/ME have been used internationally in clinical practice and research since the first was published in 1988, and these definitions have differed mainly in the minimum duration of fatigue and the type and number of additional symptoms.^7^ In the UK, guidelines for the diagnosis and management of CFS/ME were published by the National Institute for Health and Care Excellence (NICE) in 2007.^8^ These define CFS/ME as persistent and/or recurrent fatigue of ≥4 months’ duration, of new or specific onset (not lifelong), characterised by post-exertional malaise, unexplained by other conditions, and accompanied by at least one of a dozen symptoms, including sleep-wake perturbations, cognitive dysfunction, and muscle and/or joint pain. Referral to specialist CFS/ME care should be offered within six weeks for children and young people, within 3–4 months for adults with moderate symptoms, and immediately for people with severe symptoms. NHS specialist CFS/ME services offer patient-centred programmes aiming to rehabilitate patients by increasing physical, emotional, and cognitive capacities, whilst managing the impact of symptoms.^8^

By contrast, there are as yet no UK national guidelines for FM. Criteria defining FM were published in 1990 by the American Royal College of Rheumatology.^9^ These were not intended to be used in clinical practice, and the criteria were modified in 2011 to improve their clinical utility.^10^ In essence, FM should be considered in any patient reporting chronic multifocal or diffuse pain that is not explained by injury, inflammation, or other conditions.^11^ Care pathway recommendations for FM patients in the UK have only recently been published, by the British Pain Society.^12^ These focus on non-specialist (primary care) management of FM, with referral to specialist care reserved for severely affected patients who do not respond to treatment. As with CFS/ME, treatment options tend towards therapies such as Cognitive Behavioural Therapy (CBT). Pharmacological treatments approved in the USA for treatment of FM do not have UK approval for FM.^11^

Trends in the incidence of CFS/ME and FM diagnoses in the UK were last reported for the period 1990 to 2001, at the end of which there were approximately 15 CFS/ME and 35 FM cases recorded per 100,000 people.^13^ Incidence of FM increased dramatically from the mid-1990s to 2001, and CFS/ME increased from 9% to 26% of all fatigue diagnoses. Trends in the incidence of CFS/ME diagnoses might be expected to have changed since 2001, because of growing awareness and recognition of CFS/ME as a legitimate disease, and publication of NICE guidelines. The aim of the present study was to use nationally representative primary care data to investigate trends in recorded diagnoses of CFS/ME and FM in the UK between 2001 and 2013, together with trends in the incidence of diagnoses related to CFS/ME – post-viral fatigue syndrome (PVFS) and asthenia/debility – and presentation of fatigue symptoms at GP consultations. We also wanted to investigate whether primary care data would reveal variation in incidence by socioeconomic status, given conflicting evidence from an earlier study based on data from CFS/ME specialist services in England.^14^

## Methods

### Data source

The Clinical Practice Research Datalink (CPRD), formerly known as the General Practice Research Database (GPRD), is an anonymised research database aggregating medical records data from participating general practices across the UK (approximately 7% of 10,000 practices in 2012).^15^ Practices contributing to CPRD are broadly representative of general practices in the UK in terms of practice size and geographical distribution, and the source population (approximately four million ‘active’ patients, i.e. alive and registered with a GP) is broadly representative of the population of the UK in terms of age, sex, and ethnicity. GPs enter medical diagnoses and symptoms as Read codes, a hierarchical coding system used to record clinical information. Procedures, prescriptions, and referrals to secondary care are also recorded, and linkage to Hospital Episode Statistics (HES) data is available for around half of the participating practices. CPRD provides two sets of data quality criteria: ‘up-to-standard’ (UTS) time for practices and ‘acceptability’ for patients. These criteria do not ensure data quality, but the CPRD recommends that these measures are used as a first step to selecting research-quality patients and periods of quality data recording. The UTS date is a practice-based quality metric based on the continuity of recording and the number of recorded deaths. The acceptable patient metric is based on registration status, recording of events in the patient record, and valid age and gender. Patients with non-contiguous records or poor data recording, which thereby raises suspicion about the validity of that patient’s record, are excluded. For this study, data were obtained from the 660 general practices in the UK in the CPRD from 1 January 2001 to 31 December 2013 whose recording of data was judged to be UTS, in order to provide a stable denominator for calculating incidence rates. The study protocol was approved by the MHRA Independent Scientific Advisory Committee (protocol #14_041R).

### Study cohort

#### Diagnoses

Patients were identified by Read code (Supplementary Table 1) as having an event – chronic fatigue syndrome (CFS/ME), fibromyalgia (FM), post-viral fatigue syndrome (PVFS), or asthenia/debility diagnosis or referral to a CFS/ME specialist service – during the study period (1 January 2001 to 31 December 2013). The ‘index event’ was the earliest event of interest for a patient during the study period and within the practice’s up-to-standard (UTS) period and the patient’s UTS registration period. Patients were required to have at least 12 months of UTS data prior to the index event. For the purpose of estimating incidence rates, we considered incident, i.e. ‘new’, diagnoses to be those index events for which there were no preceding diagnosis of CFS/ME, FM, PVFS, or asthenia/debility in the patient’s Clinical Practice Research Datalink (CPRD) medical record. Read codes for referral to specialist services were introduced in 2010. Diagnoses which were made after a referral and which occurred within the patient’s UTS period (and for which there was no prior diagnosis) were treated as incident diagnoses.

#### Fatigue symptoms

Patients were identified as having presented with fatigue if they had one or more events (per calendar year) with a Read code for a fatigue symptom during the study period (Supplementary Table 1). Events must have occurred during the patient’s registration period and during the practice UTS period, and patients were required to have at least 12 months of UTS data prior to the event.

#### Practice-level socioeconomic status

The Index of Multiple Deprivation (IMD) score was used as a measure of socioeconomic status for the practice, based on its postcode. The IMD is the UK government’s official measure of deprivation.^16^ It is a composite score derived from seven domains: income, employment, health and disability, education skills and training, barriers to housing and services, crime and disorder, and living environment.

### Statistical analyses

Annual incidence rates (and 95% confidence intervals (CIs)) of diagnoses and fatigue symptoms were calculated by summing the number of events during each calendar year per practice, dividing by the number of acceptable patients registered during the corresponding year, and then calculating an overall rate and 95% CI per 100,000 patients. ‘Lewis plots’ were used to verify that requiring patients to have at least 12 months of UTS data prior to the index event had removed the excess events, which tend to occur shortly after a patient has registered with a practice.^17^ Trends were analysed using join point (segmented) regression software (Joinpoint Regression Program, Version 4.3.1.0 – April 2016; Statistical Methodology and Applications Branch, Surveillance Research Program, National Cancer Institute), which performs segmented regression to estimate the annual percent change (APC) in incidence rates and the number and location of join points (points at which trends change).^18^ Join point software performs pairwise comparisons of models differing by one join point to determine the model with optimum fit to the data series. We allowed a maximum of three join points (because we had 13 data points in our analysis), and an overall significance level of 5% was adopted for the comparisons of models applied to each data series. The models incorporate variation using the standard error of the rate; annual percentage charge for each segment is estimated by fitting a regression line to the natural logarithm of the rate, using calendar year as an independent variable.^19^ We calculated incidence rates stratified by sex, age group, and IMD quintile, and we used multivariable negative binomial regression to estimate adjusted incidence rate ratios (IRR) with sex, age, IMD quintile, and calendar year as independent variables, and robust standard errors to account for clustering within practices. We included interaction terms to test for evidence of interaction between age, sex, and IMD quintile with time, using calendar year as a continuous linear variable if join point had indicated a constant trend (no join point) or using time periods corresponding to the segments between join points. Negative binomial regression models were fitted using Stata (StataCorp. 2015. Stata Statistical Software: Release 14. College Station, TX: StataCorp LP). We also used the Joinpoint regression software’s implementation of a statistical test for parallelism to test for differences in trends by age, sex, and IMD quintile, using sex = female, age = 40–49 years, and IMD = middle quintile (Q3) as reference level.^20^

## Results

### Diagnoses and referral events

**Figure 1.**
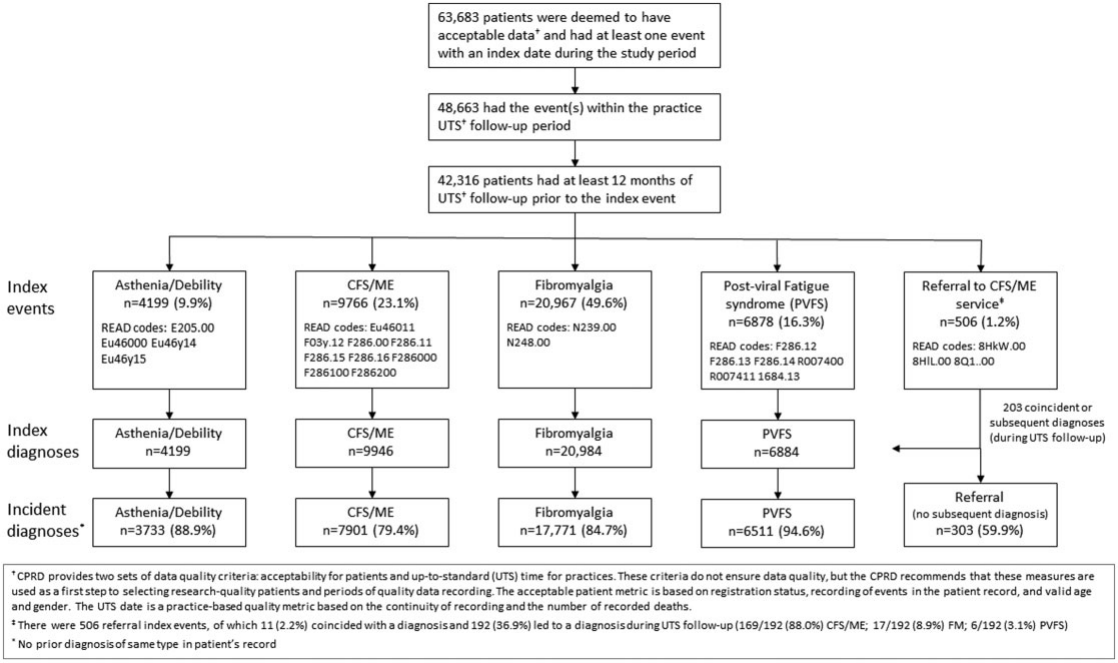
CPRD patient inclusion flowchart for this study.

From the source population in CPRD, 63,683 patients were deemed to have acceptable data and had at least one diagnostic index event during the study period. Of these patients, 48,663 had the event(s) within the practice UTS period, of whom 42,316 patients had at least 12 months of UTS data prior to the index date. FM accounted for half of the diagnoses (49.6%), followed by CFS/ME (23.1%), PVFS (16.3%), and asthenia/debility (9.9%) (Figure 1).

There were 1196 referrals (for 933 patients) recorded since the relevant Read codes for referral to specialist CFS/ME services were introduced (2010), of which 506 were index events. Of these, 11 (2.2%) coincided with a CFS/ME diagnosis and 192 (36.9%) led to a diagnosis during the patient’s UTS period: 169/192 (88.0%) CFS/ME; 17/192 (8.9%) FM; and 6/192 (3.1%) PVFS (Figure 1).

Diagnoses and referrals, preceding events (during the first 12 months of the UTS period or predating the start of the UTS period), and time between earliest preceding event (if any) and the index diagnosis or referral, are summarised in Table 1. Overall, 81.9% (34,677/42,316) of patients had no previously recorded diagnosis of CFS/ME, PVFS, A/D, or FM or referral to CFS/ME service in their medical record, and 12.5% (5276/42,316) had a previous event of the same type as the index event. The remaining 2363 patients (5.6%) had at least one prior event of a different type to the index event. Lewis plots indicated a constant rate of diagnoses from the 13th month of the UTS period onwards (Supplementary Figure 1).

**Table 1.** Index events (diagnoses and referrals recorded during the study period, 2001–2013).^a^

		Preceding events						
Index event (diagnosis or referral)	Frequency	None	Asthenia/Debility only	CFS/ME only	FM only	PVFS only	Referral only	Any^b^
Asthenia and debility	4199	3699 (88.1%)	459 (10.9%)	10 (0.2%)	6 (0.1%)	18 (0.4%)	0 (0.0%)	7 (0.2%)
Time from earliest preceding event to index event, median (IQR) years			5 (3–8)	5 (3–9)	6 (4–8)	9 (6–14)	–	7 (5–12)
CFS/ME	9946	7199 (72.4%)	56 (0.6%)	1556 (15.6%)	67 (0.7%)	388 (3.9%)	7 (0.1%)	673 (6.8%)
Time from earliest preceding event to index event, median (IQR) years			10 (6–14)	5 (2–10)	5 (3–7)	9 (4–13)	1 (0–10)	6 (1–11)
Fibromyalgia (FM)	20,984	17,157 (81.8%)	134 (0.6%)	241 (1.1%)	2947 (14.0%)	176 (0.8%)	0 (0.0%)	329 (1.6%)
Time from earliest preceding event to index event, median (IQR) years			11 (8–15)	7 (2–12)	4 (2–8)	12 (8–16)	–	8 (4–12)
Post-viral fatigue syndrome (PVFS)	6884	6368 (92.5%)	28 (0.4%)	77 (1.1%)	29 (0.4%)	311 (4.5%)	0 (0.0%)	71 (1.0%)
Time from earliest preceding event to index event, median (IQR) years			10 (4–13)	6 (3–13)	8 (6–11)	8 (3–13)	–	5 (2–12)
Referral to CFS/ME service	303	254 (83.8%)	1 (0.3%)	30 (9.9%)	3 (1.0%)	3 (1.0%)	3 (1.0%)	9 (3.0%)
Time from earliest preceding event to index event, median (IQR) years			16	8 (4–15)	10 (3–20)	24 (16–26)	1 (0–2)	4 (2–6)

a For the purpose of estimating incidence rates, we considered incident diagnoses to be those index events for which there was no preceding diagnosis of CFS/ME, FM, PVFS, or asthenia/debility in the patient’s CPRD medical record (Preceding events = ‘None’). There were 506 referral index events, of which 11 (2.2%) coincided with a diagnosis and 192 (36.9%) led to a diagnosis during UTS follow-up.

b Any other sequence of two or more events (asthenia/debility, CFS/ME, FM, PVFS, referral to CFS/ME service).

### Trends in the incidence of diagnoses

**Figure 2.**
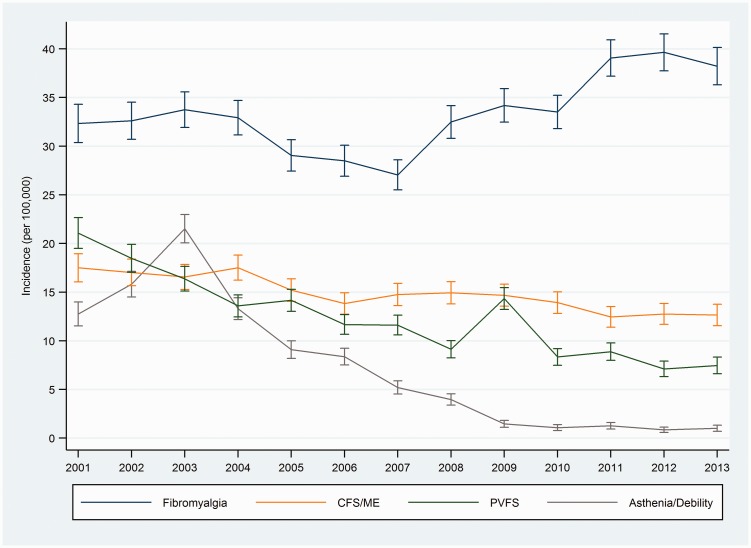
Trends in recorded diagnoses of chronic fatigue syndrome (CFS/ME), fibromyalgia, post-viral fatigue syndrome (PVFS), and asthenia/debility (2001–2013) (vertical bars indicate 95% CI).

The average annual incidence of recorded cases of CFS/ME over the whole study period was 14.8 (95% CI 14.5, 15.1) per 100,000 people; for FM, the annual rate per 100,000 people was 33.3 (32.8, 33.8); for PVFS 12.2 (11.9, 12.5), and for asthenia/debility 7.0 (6.8, 7.2). Trends in incidence rates of diagnoses are illustrated in Figure 2 (see Supplementary Table 2 for incidence rates). Annual incidence of CFS/ME decreased from 17.5 (16.1, 18.9) in 2001 to 12.6 (11.5, 13.8) in 2013. Annual incidence of FM decreased from 32.3 (30.4, 34.3) in 2001 to 27.1 (25.5, 28.6) in 2007, and then increased to 38.2 (36.3, 40.1) per 100,000 people in 2013. CFS/ME as a percentage of all fatigue diagnoses (excluding FM) increased from 31.1% in 2001 to 59.9% in 2013, whilst asthenia/debility declined from 24.8% to 4.8% and PVFS declined from 41.1% to 35.4% (Supplementary Figure 2).

Join point regression analysis indicated constant (no join point) trends over the study period (2001–2013) as having the best fit to CFS/ME and PVFS incidence rates. The annual percent change for CFS/ME was −2.8% (95% CI −3.6%, −2.0%) and −7.8% (−10.1%, −5.5%) for PVFS (Table 2). Trends in diagnoses of asthenia/debility and FM each had a best fitted model with one join point: asthenia/debility diagnoses increased to 2003 and then declined steeply, at −29.6% (−33.9%, −24.9%) per annum; FM diagnoses decreased to 2007 (APC −2.4% (−5.3%, 0.7%)) and then increased by 5.9% (3.1%, 8.8%) per annum to 2013.

**Table 2.** Trends in incidence of diagnoses and fatigue symptoms.

Diagnosis/symptoms	Period 1	Annual percent change (95% CI)	Period 2	Annual percent change (95% CI)	Period 3	Annual percent change (95% CI)
Asthenia and debility	2001–2003	27.2 (−14.8, 90.0)	2003–2013	−29.6 (−33.9, −24.9)^a^		
CFS/ME	2001–2013	−2.8 (−3.6, −2.0)^a^				
Fibromyalgia	2001–2007	−2.4 (−5.3, 0.7)	2007–2013	5.9 (3.1, 8.8)^a^		
Post-viral fatigue syndrome (PVFS)	2001–2013	−7.8 (−10.1, −5.5)				
Fatigue symptoms	2001–2004	5.3 (1.1, 9.6)^a^	2004–2009	1.0 (−1.1, 3.3)	2009–2013	−1.7 (−3.9, 0.5)

Evidence that annual percent change is greater than or less than zero at *α* = 0.05.

### Trends in the incidence of fatigue symptoms

**Figure 3.**
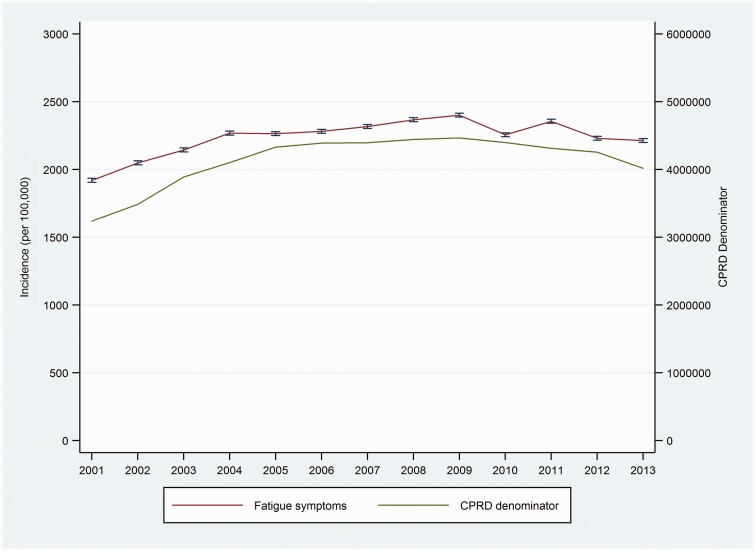
Trends in fatigue symptoms (2001–2013) (vertical bars indicate 95% CI).

Overall annual incidence of recorded fatigue symptoms was 2246 (2242 to 2250) per 100,000 people (Figure 3). The incidence of recorded fatigue symptoms increased from 2001 to 2004 (APC 5.3% (1.1%, 9.6%)) (Table 2), with the trends in the two subsequent intervals having confidence intervals which do not exclude a constant rate (APC = 0 at *α* = 0.05).

### Diagnoses and symptoms by age, sex, and practice-level socioeconomic status

**Figure 4.**
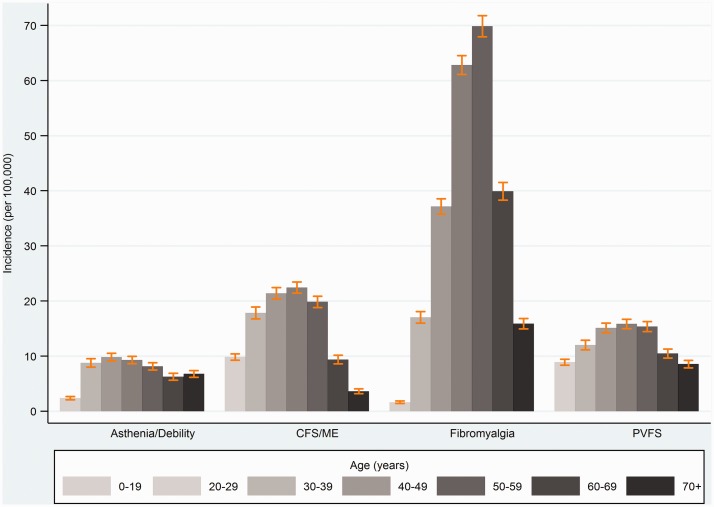
Recorded diagnoses of chronic fatigue syndrome (CFS/ME), fibromyalgia, post-viral fatigue syndrome (PVFS), and asthenia/debility by age (vertical bars indicate 95% CI).

**Figure 5.**
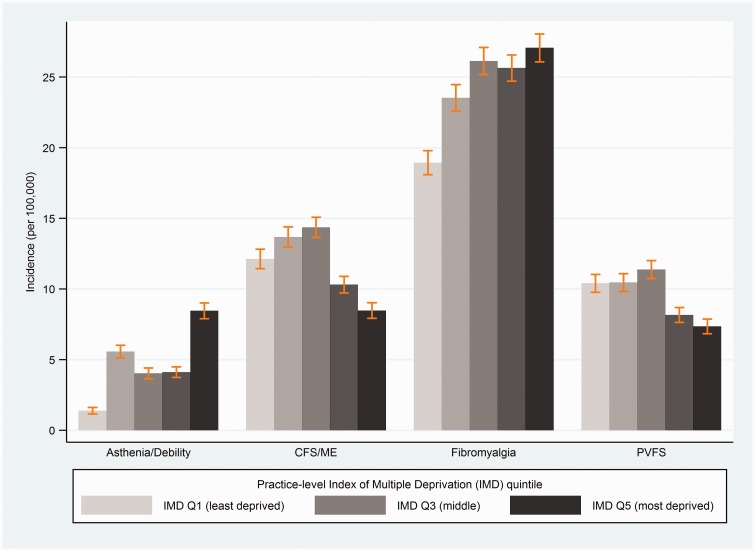
Recorded diagnoses of chronic fatigue syndrome (CFS/ME), fibromyalgia, post-viral fatigue syndrome (PVFS), and asthenia/debility by Index of Multiple Deprivation (IMD) quintile (vertical bars indicate 95% CI).

All diagnoses showed strong evidence of variation by age and sex, and all except asthenia/debility showed evidence of variation across practice-level quintiles socioeconomic status (Table 3). Incidence rates were higher amongst women, and tended to peak between ages 30–59 years (Figure 4). Incidence rates of CFS/ME were 2.4-fold higher among women (incidence rate ratio (IRR) 2.35 (95% CI 2.19, 2.53)), with peak incidence in the 40–49 years age group (IRR 1.64 (1.48, 1.83), compared with <20 years age group). Women had six-fold higher incidence of FM (IRR 6.13 (5.50, 6.82)), peaking between ages 40 and 49 years (IRR 25.3 (20.6, 31.1), compared with ages <20 years). Social patterns were in the opposite directions for CFS/ME and FM, with CFS/ME incidence being lowest and FM incidence tending to be higher in the two most deprived IMD quintiles (Figure 5). The incidence rate ratios comparing the bottom (most deprived) versus the top (least deprived) IMD quintile were IRR 0.61 (0.50, 0.75) for CFS/ME, IRR 1.40 (0.95, 2.06) for FM (Table 3).

**Table 3.** Incidence rate ratios (IRR (95% CI)) for diagnoses, referrals, and symptoms (by age, sex, and practice-level socioeconomic status).

		Asthenia/Debility	CFS/ME	FM	PVFS	Referral	Fatigue (symptom)
Female (cf. male)		2.15 (1.94, 2.38)	2.35 (2.19, 2.53)	6.13 (5.50, 6.82)	2.01 (1.89, 2.14)	2.09 (1.48, 2.95)	2.28 (2.26, 2.31)
Age (cf. <20) years	20 to 29	2.16 (1.66, 2.83)	0.86 (0.76, 0.96)	4.86 (4.02, 5.89)	0.66 (0.58, 0.75)	2.39 (1.43, 4.02)	2.41 (2.34, 2.48)
	30 to 39	3.46 (2.68, 4.46)	1.39 (1.24, 1.55)	13.5 (11.0, 16.7)	1.01 (0.90, 1.14)	2.56 (1.39, 4.72)	2.33 (2.26, 2.40)
	40 to 49	4.01 (3.03, 5.30)	1.64 (1.48, 1.83)	25.3 (20.6, 31.1)	1.22 (1.08, 1.38)	3.07 (1.82, 5.18)	2.38 (2.31, 2.46)
	50 to 59	2.80 (2.10, 3.74)	1.31 (1.17, 1.46)	24.7 (20.2, 30.4)	0.99 (0.88, 1.11)	1.43 (0.85, 2.40)	2.47 (2.39, 2.54)
	60 to 69	1.27 (1.02, 1.58)	0.56 (0.48, 0.65)	12.6 (10.3, 15.3)	0.57 (0.50, 0.66)	0.66 (0.32, 1.38)	2.49 (2.41, 2.57)
	70+	1.91 (1.47, 2.47)	0.23 (0.19, 0.27)	5.99 (4.88, 7.36)	0.51 (0.44, 0.60)	0.28 (0.12, 0.64)	3.51 (3.41, 3.62)
Practice-level IMD quintile (cf. Least deprived)^a^	Quintile 2	0.59 (0.17, 2.04)	1.00 (0.85, 1.17)	1.21 (0.94, 1.57)	0.91 (0.66, 1.25)	1.63 (0.75, 3.52)	0.98 (0.86, 1.10)
	Quintile 3	1.09 (0.30, 4.03)	1.04 (0.85, 1.26)	1.05 (0.86, 1.27)	0.80 (0.57, 1.13)	1.90 (0.87, 4.16)	0.87 (0.79, 0.96)
	Quintile 4	0.97 (0.33, 2.80)	0.82 (0.69, 0.98)	1.24 (1.01, 1.51)	0.66 (0.50, 0.87)	1.37 (0.66, 2.85)	0.85 (0.77, 0.94)
	Most deprived	2.30 (0.72, 7.38)	0.61 (0.50, 0.75)	1.40 (0.95, 2.06)	0.67 (0.48, 0.93)	0.97 (0.47, 2.02)	0.96 (0.86, 1.07)

a Practice-level socioeconomic status was measured by its Index of Multiple Deprivation (IMD) score, divided into quintiles. IRR are adjusted for all of the factors shown in the table.

**Figure 6.**
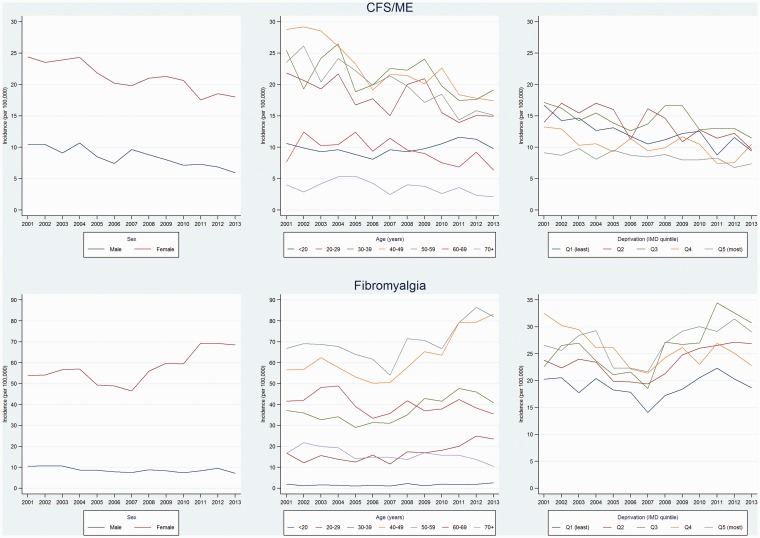
Trends in recorded diagnoses of chronic fatigue syndrome (CFS/ME) and fibromyalgia (2001–2013) by sex, age, and Index of Multiple Deprivation (IMD) quintile.

There were no interactions (all *p* ≥ 0.2) between age, sex, IMD, and CFS/ME diagnostic incidence. However, there was weak evidence (test for parallelism *p* ≤ 0.1) of a less steep downward trend in CFS/ME incidence in male compared with female patients, and in age groups 20–29 years and 30–39 years compared with 40–49 years (Figure 6, Supplementary Table 3). CFS/ME incidence in patients age < 20 years followed a different trend to other age groups (*p* < 0.001), decreasing from 2001 to 2006, then increasing from 2006 to 2011. There was strong evidence of interaction (*p* < 0.001) between age and FM incidence and between sex and FM incidence, but there was no interaction with IMD (*p* = 0.2). The pattern of a pre-2007 decrease followed by a post-2007 increase was evident only among female patients, with male patients instead showing a steady decline (test for parallelism *p* = 0.002) (Figure 6, Supplementary Table 4). Similarly, the pre-/post-2007 pattern was apparent among patients in the 30–39, 40–49, and 50–59 year age groups, whereas there was a constant increase (no join point) in incidence in patients aged <20 and 20–29 years and a constant decrease in incidence in patients age 60–69 and 70+ years.

## Discussion

### Statement of principal findings

Our study has shown that the incidence of CFS/ME diagnoses declined over the period 2001–2013, whereas FM diagnoses (after an initial decline) showed an overall increase. Asthenia and debility became almost extinct as diagnostic labels, having begun the decade on a par with CFS/ME. Diagnoses of post-viral fatigue syndrome (PVFS) also fell steeply, to one-third of the incidence in 2001. Clearly, the decline in asthenia/debility and PVFS diagnoses did not translate into an increase in CFS/ME diagnoses. We had anticipated a change in overall trends in CFS/ME diagnoses after the publication of NICE guidelines in 2007, but none was apparent (although cases in patients <20 years old increased from 2006 to 2011). FM diagnoses increased markedly from 2007 onwards, levelling off from 2011 to 2013 at an annual incidence rate of 40 per 100,000. This is roughly three times higher than the incidence of CFS/ME. As expected, incidence rates of CFS/ME and FM were higher amongst women and peaked between ages 30 and 59 years. Incidence of CFS/ME showed a strong social gradient, with lowest incidence in the bottom (most deprived) socioeconomic quintile, whereas FM had lower incidence in the top (least deprived) quintile.

### Strengths and limitations of the study

The main strength of our study is that it uses CPRD data, comprising the medical records of approximately four million ‘active’ patients (alive and registered with a GP), or 7% of the UK population.^15^ CPRD patients are representative of the UK population, and the data are subject to external and internal quality control. Active patients have a median of 9.4 years (IQR 3.4–13.9) of data,^15^ which gives us some (but not absolute) confidence in classifying diagnoses as ‘incident’ if the patient’s medical record has no previously recorded diagnoses.

One limitation of our study is that we examined recorded data rather than actual incidence, i.e. we are describing incidence rates of GPs’ recording of diagnostic codes. This will have missed cases not recognised by the GP, recognised but not entered into the clinical records, and recognised but entered into the records as free text. Another limitation is that diagnoses were not independently validated. We know from studies in specialist services that CFS/ME is frequently misdiagnosed in primary care.^21^ In 2005, 48% of GPs in one English region did not feel confident about making a diagnosis of CFS/ME, and 28% did not recognise CFS/ME as a legitimate illness.^22^ Although knowledge and awareness of CFS/ME in primary care had improved towards the end of our study period,^23^ non-specialist clinicians still experienced difficulty and lack of confidence in making a diagnosis of CFS/ME.^24^

Our data do not tell us whether diagnoses were made by GPs, or recorded in patients’ medical records following a specialist consultation. Read codes for referral to specialist services were only introduced in 2010, limiting our ability to explore this aspect of the CFS/ME diagnostic pathway. However, our estimated incidence of new CFS/ME diagnoses for the period 2008–2010 (in 2009, 14.5 (95% CI 13.6 to 15.8) per 100,000) is not inconsistent with estimates based on data from NHS specialist CFS/ME services in England (in 2009, 22 (95% CI 17 to 29) per 100,000), if we consider that the latter figure includes re-referrals.^14^ Incidence of CFS/ME diagnoses in 2009 from our data, including patients who had a previously recorded CFS/ME diagnosis, is 17.0 (95% CI 15.8 to 18.3). This consistency with estimates from clinical services would suggest that the majority of patients who have a CFS/ME diagnosis recorded by their GP have had this diagnosis made or confirmed by a specialist service. Our overall annual incidence rate estimate of 14.8 (95% CI 14.5 to 15.1) per 100,000 is also consistent with a rate of 15 (95% CI 6 to 41) per 100,000 reported for the period 2007–2010 based on data from a primary care cross-sectional study in three English regions.^25^ These comparisons suggest that we can have reasonable confidence in the validity of CFS/ME diagnoses in CPRD records. During the early part of our study period, trends in fatigue symptoms appeared to parallel the underlying CPRD population denominator. This suggests a degree of artefact, for which we could find no explanation. Similarly, the two ‘spikes’ in asthenia/debility (in 2003) and PVFS (in 2009) are possibly artefactual, because we are not aware of any drives to enhance entry of these codes on GP systems. One possible explanation for the spike in PVFS cases is the 2009 flu pandemic, which started in May 2009 and continued in waves until the summer of 2010.^26^

### Our study in relation to other studies

The overall decline in ‘fatigue’ diagnoses (CFS/ME, PVFS, and asthenia/debility) continues the downward trend (a 44% decrease) reported for the period 1990–2001 using data from the same source (then called the General Practice Research Database (GPRD)).^13^ As in our study, this trend was set against a backdrop of no overall change in incidence of fatigue symptoms. This earlier study also showed the emergence in the mid 1990s of fibromyalgia as a diagnosis, which the authors ascribed to fashions in diagnostic labelling rather than to a true increase in incidence.^13^ In addition to publication of NICE guidelines, the period covered by our study witnessed a growth in NHS specialist CFS/ME service provision across England,^27^ such that 140 of 152 Primary Care Trusts (PCTs) had commissioned a specialist service by 2010 (PCTs were abolished in 2013 during a reorganisation of the NHS).^14^ In the context of these changes, the steady decline in CFS/ME diagnoses is perplexing. In the absence of curative treatments and, given that we have no reason to suspect underlying trends in the causal agents and risk factors for CFS/ME (or PVFS), we cannot discount a trend in diagnostic labelling. However, the decline does not show any sudden fluctuations and does not correlate with declines in alternative diagnoses (PVFS and asthenia/debility), suggesting that it may reflect a real trend.

Although specialist service provision for paediatric CFS/ME in the UK is geographically much less comprehensive than for adults, with many barriers to accessing the few available services,^28^ the prognosis for natural recovery in children and young people is better than in adults.^29^ Also, guidelines for the management of paediatric CFS/ME were published by the Royal College of Paediatrics and Child Health in 2004.^30^ We note that the observed annual incidence for patients <20 years of age (10 per 100,000) translates into a prevalence of 1–2% (if we assume an average 12–24 months’ duration of illness), which is entirely consistent with paediatric CFS/ME prevalence estimates from population-based studies.^31^

We would expect that a substantial proportion (if not all) PVFS cases should have been classified as CFS/ME. Viral infections are known to trigger CFS/ME,^32^ and fatigue of ≥4 months’ duration after the acute phase of an infection has passed should, according to NICE criteria, warrant consideration for a diagnosis of CFS/ME.^8^ Towards the end of our study period, this reclassification would increase the incidence of CFS/ME by around 50%, but the trend in CFS/ME diagnoses would still be downwards. Population-wide incidence of CFS/ME in Norway from 2008 to 2012 as indicated by ICD-10 code G93.3 (‘post-viral fatigue syndrome/benign myalgic encephalomyelitis’) was 25.8 (25.2, 26.5) per 100,000 person years.^33^ This is 74% higher than the incidence in our study, possibly because the ICD-10 code combines PVFS and CFS/ME in a single classification.

Whether the levelling out of incident FM diagnoses towards the end of our study period represents a plateau reflecting true incidence remains to be seen. The three-fold higher incidence of FM compared with CFS/ME is supported by prevalence estimates, which show that the prevalence of FM (1–5%, depending on classification criteria)^34^ is several times higher than the prevalence of CFS/ME (0.8% (95% CI 0.2% to 1.3%) from a meta-analysis of prevalence studies based on clinically confirmed cases).^35^ In adults, both are long-term conditions with poor prognosis and limited therapeutic options.^36,37^ The evidence base for CFS/ME therapies is arguably stronger than for FM,^1^ but a range of pharmacological agents are available (albeit unsupported by strong evidence of effect) for symptom control in FM.^11,38^ These include three drugs approved in the USA during the period of our study (pregabalin in 2007, duloxetine in 2008, and milnacipran in 2009). The absence of UK approval for these drugs means that we cannot ascribe the post-2007 increase in FM incidence to GP’s being more willing to diagnose FM because of the availability of prescription drugs for FM.

The age and sex distributions of CFS/ME and FM are well-documented,^1,3,33,34^ socioeconomic variation less so. We know that childhood social adversity increases the risk of CFS/ME in children and adolescents,^31^ and that this increased risk may persist into adulthood.^39^ Evidence for a link between social adversity in adulthood and risk of CFS/ME is perhaps less convincing, more likely to be confounded by variation in risk, e.g. across ethnic groups, and more susceptible to selection bias, e.g. by access to healthcare, but the overall pattern suggests higher risk among lower socioeconomic groups. In an analysis of patient postcode data from seven CFS/ME specialist services in England, we showed that assessment rates in three of the services were 39–44% lower in the bottom (most deprived) compared with the top (least deprived) IMD quartiles,^14^ which is consistent with the 39% lower diagnostic incidence that we reported, comparing the bottom with the top IMD quintile. There was no social pattern in the other four services, a discrepancy which we attributed partly to variation in the ethnic minority composition of the communities served by the services. Obstacles to accessing specialist care provide the most likely explanation for decreasing CFS/ME diagnostic incidence with increasing social deprivation, if we assume that the well-documented difficulties experienced by people seeking diagnosis and/or specialist treatment for CFS/ME are more acute for people who have limited resources.^6,23,28^

That FM diagnoses showed the opposite trend requires a different explanation, although we note that the social pattern was for the top (least deprived) quintile to have lower incidence than the other four quintiles, each of which had similar incidence with only weak evidence for the bottom (most deprived) quintile having the highest incidence. As with CFS/ME, evidence suggests higher risk of pain disorders such as FM among lower social classes.^40,41^ One possible explanation for the different pattern seen for FM compared with CFS/ME could be that FM is more likely to be diagnosed and managed within the primary care setting; not because GPs are more confident about diagnosing and managing FM, but because there are no specialist services. Indeed, the British Pain Society care pathway recommends primary care management of FM, with referral to specialist care only for severely affected patients who do not respond to treatment.^12^ Another explanation could be that GPs respond more decisively to the defining symptom of FM (pain) in patients who are likely to be presenting with other symptoms and co-morbidities,^40^ whereas the cardinal symptoms of CFS/ME present a more complex and less-specific diagnostic picture, further complicated by CFS/ME-related co-morbidities such as pain and mood disorders.^4^ This argument is strengthened by the observation that patients with FM and lower socioeconomic status reported greater symptom severity and functional impairment,^42^ although this pattern has also been observed in community-based samples of people diagnosed with CFS/ME.^43^

### Unanswered questions and future research

The strong evidence for a social gradient in CFS/ME diagnoses, contrary to evidence indicating that incidence should if anything be higher at lower socioeconomic levels, raises a question which can perhaps only be answered by collecting prospective data at primary care level, supplemented by qualitative research into the diagnostic and referral process for patients across diverse social and ethnic groups. Most CFS/ME specialist services report anecdotally that black and minority ethnic groups are underrepresented among their service users, despite CFS/ME possibly being more prevalent in UK ethnic minorities,^44^ but the extent to which this disparity reflects differences in cultural norms or professional practice is unknown.^45^ This aspect of CFS/ME care provision requires investigation, something which should become possible as collection of ethnicity data within CPRD improves.^46^

## Conclusions

Our study has provided an up-to-date picture of trends in the incidence of CFS/ME and FM diagnoses, indicating an ongoing need to provide care pathways and commission specialist services for these relatively common and debilitating long-term conditions. In addressing this need, two salient features of our findings need to be taken into account, namely: (1) that the incidence of FM is three times higher than incidence of CFS/ME, yet there are no national guidelines for the diagnosis and management of FM and (2) that the incidence of CFS/ME diagnoses appears to be 40% lower in the most versus least deprived socioeconomic quintile, despite evidence that CFS/ME should be as common, if not more common, among people experiencing higher levels of social adversity.

## Supplementary Material

Supplementary material

Supplementary material

## Declarations

### Guarantor

SMC.

### Provenance

Not commissioned; peer-reviewed by Sanjeev Kumar, Vasiliy Vlassov, and Julie Morris.
