# Supplementary material for: Trends in the incidence of chronic fatigue syndrome and fibromyalgia in the UK, 2001–2013: a Clinical Practice Research Datalink study
Source: J R Soc Med. 2017 Mar 30;110(6):231–44. doi: 10.1177/0141076817702530 (PMC5499564; doi:10.1177/0141076817702530)
Supplement: Supplementary material [file JRS702530_supplementary_tables.pdf]

Supplementary Table 1: READ codes defining diagnoses and symptoms

READ CODE	READ TERM	Type of event
Eu46011	[X]FATIGUE SYNDROME	CFS/ME
F03y.12	MYALGIC ENCEPHALOMYELITIS	CFS/ME
F286.00	CHRONIC FATIGUE SYNDROME	CFS/ME
F286.11	CFS - CHRONIC FATIGUE SYNDROME	CFS/ME
F286.15	MYALGIC ENCEPHALOMYELITIS	CFS/ME
F286.16	ME - MYALGIC ENCEPHALOMYELITIS	CFS/ME
F286000	Mild chronic fatigue syndrome	CFS/ME
F286100	Moderate chronic fatigue syndrome	CFS/ME
F286200	Severe chronic fatigue syndrome	CFS/ME
F286.12	POSTVIRAL FATIGUE SYNDROME	PVFS
F286.13	PVFS - POSTVIRAL FATIGUE SYN	PVFS
F286.14	POST-VIRAL FATIGUE SYNDROME	PVFS
R007400	[D]POSTVIRAL (ASTHENIC) SYNDROME	PVFS
1684.13	C/O - POSTVIRAL SYNDROME	PVFS
R007411	[D]POST VIRAL DEBILITY	PVFS
Eu46000	[X]NEURASTHENIA	Asthenia/Debility
Eu46y14	[X]PSYCHASTHENIA	Asthenia/Debility
Eu46y15	[X]PSYCHASTHENIA NEUROSIS	Asthenia/Debility
E205.00	NEURASTHENIA - NERVOUS DEBILITY	Asthenia/Debility
N239.00	FIBROMYALGIA	FM
N248.00	FIBROMYALGIA	FM
8HkW.00	Referral to chronic fatigue syndrome specialist team	Referral
8HlL.00	Referral for chronic fatigue syndrome activity management	Referral
8Q1..00	Activity management for chronic fatigue syndrome	Referral
R007200	[D]ASTHENIA NOS	Symptom
R202.00	[D]SENILE ASTHENIA	Symptom
R2y3.00	[D]DEBILITY, UNSPECIFIED	Symptom
168..00	TIREDNESS SYMPTOM	Symptom
168..11	FATIGUE - SYMPTOM	Symptom
168..12	LETHARGY - SYMPTOM	Symptom
168..13	MALAISE - SYMPTOM	Symptom
1682.00	FATIGUE	Symptom
1683.00	TIRED ALL THE TIME	Symptom
1683.11	C/O - "TIRED ALL THE TIME"	Symptom
1684.00	MALAISE/LETHARGY	Symptom
1684.11	C/O - DEBILITY - MALAISE	Symptom
168Z.00	TIREDNESS SYMPTOM NOS	Symptom
E205.12	TIRED ALL THE TIME	Symptom
R007.00	[D]MALAISE AND FATIGUE	Symptom
R007000	[D]MALAISE	Symptom
R007100	[D]FATIGUE	Symptom
R007211	[D]GENERAL WEAKNESS	Symptom
R007300	[D]LETHARGY	Symptom
R007500	[D]TIREDNESS	Symptom
R007z00	[D]MALAISE AND FATIGUE NOS	Symptom

Supplementary Table 2: Diagnostic events and rates (per 100,000), 2001-2013

		Asthenia/Debility		CFS/ME		Fibromyalgia		PVFS	
Year	Denominator	Events	Rate (95% CI)	Events	Rate (95% CI)	Events	Rate (95% CI)	Events	Rate (95% CI)
2001	3222694	411	12.8 (11.5, 14.0)	564	17.5 (16.1, 18.9)	1042	32.3 (30.4, 34.3)	679	21.1 (19.5, 22.7)
2002	3465374	548	15.8 (14.5, 17.1)	590	17.0 (15.7, 18.4)	1130	32.6 (30.7, 34.5)	640	18.5 (17.0, 19.9)
2003	3865675	832	21.5 (20.1, 23.0)	640	16.6 (15.3, 17.8)	1305	33.8 (31.9, 35.6)	633	16.4 (15.1, 17.7)
2004	4075970	542	13.3 (12.2, 14.4)	714	17.5 (16.2, 18.8)	1342	32.9 (31.2, 34.7)	554	13.6 (12.5, 14.7)
2005	4301541	391	9.1 (8.2, 10.0)	654	15.2 (14.0, 16.4)	1250	29.1 (27.4, 30.7)	609	14.2 (13.0, 15.3)
2006	4361284	365	8.4 (7.5, 9.2)	603	13.8 (12.7, 14.9)	1243	28.5 (26.9, 30.1)	509	11.7 (10.7, 12.7)
2007	4365288	227	5.2 (4.5, 5.9)	644	14.8 (13.6, 15.9)	1181	27.1 (25.5, 28.6)	507	11.6 (10.6, 12.6)
2008	4414083	175	4.0 (3.4, 4.6)	659	14.9 (13.8, 16.1)	1434	32.5 (30.8, 34.2)	403	9.1 (8.2, 10.0)
2009	4434175	65	1.5 (1.1, 1.8)	651	14.7 (13.6, 15.8)	1516	34.2 (32.5, 35.9)	636	14.3 (13.2, 15.5)
2010	4367562	47	1.1 (0.8, 1.4)	608	13.9 (12.8, 15.0)	1464	33.5 (31.8, 35.2)	364	8.3 (7.5, 9.2)
2011	4280588	54	1.3 (0.9, 1.6)	533	12.5 (11.4, 13.5)	1672	39.1 (37.2, 40.9)	380	8.9 (8.0, 9.8)
2012	4217212	36	0.9 (0.6, 1.1)	538	12.8 (11.7, 13.8)	1672	39.6 (37.7, 41.5)	300	7.1 (6.3, 7.9)
2013	3976481	40	1.0 (0.7, 1.3)	503	12.6 (11.5, 13.8)	1520	38.2 (36.3, 40.1)	297	7.5 (6.6, 8.3)

Supplementary Table 3: Comparisons of trends in incidence of CFS/ME diagnoses by age, sex and IMD quintile†

		Period 1	Annual Percent Change (95% CI)	Period 2	Annual Percent Change (95% CI)	Period 3	Annual Percent Change (95% CI)	Test for parallelism
Sex	Female	2001-2013	-4.0 (-5.5, -2.4)*					Reference
	Male	2001-2013	-2.6 (-3.4, -1.7)*					P=0.04
Age (years)	<20	2001-2006	-4.3 (-7.2, 1-.3)*	2006-2011	6.4 (2.3, 10.7)*	2011-2013	-6.5 (-17.5, 6.0)	P=0.001
	20 to 29	2001-2013	-2.9 (-4.9, --0.9)*					P=0.10
	30 to 39	2001-2013	-2.1 (-4.1, -0.1)*					P=0.04
	40 to 49	2001-2013	-4.2 (-5.5, -2.9)*					Reference
	50 to 59	2001-2013	-4.2 (-5.5, -2.9)*					P=0.99
	60 to 69	2001-2013	-3.5 (-6.3, -0.6)*					P=0.60
	70+	2001-2013	-4.6 (-8.8, -0.2)*					P=0.82
Practice-level IMD quintile	Least deprived	2001-2013	-3.9 (-5.3, -2.5)*					P=0.37
	Quintile 2	2001-2013	-3.2 (-5.5, -0.8)*					P=0.72
	Quintile 3	2001-2013	-2.9 (-4.9, -0.8)*					Reference
	Quintile 4	2001-2013	-2.0 (-4.0, -0.1)*					P=0.54
	Most deprived	2001-2013	-2.0 (-3.2, -0.9)*					P=0.40

† Trends shown are best fit to data for individual levels of each categorical variable. Test for parallelism P-value indicates strength of evidence for departure from parallel trend with reference level of categorical variable

* Evidence that Annual Percentage Change is greater than or less than zero at α=0.05.

Supplementary Table 4: Comparisons of trends in incidence of FM diagnoses by age, sex and IMD quintile[†]

		Period 1	Annual Percent Change (95% CI)	Period 2	Annual Percent Change (95% CI)	Period 3	Annual Percent Change (95% CI)	Test for parallelism
Sex	Female	2001-2007	-1.7 (-4.8, 1.4)	2007-2013	6.4 (3.6, 9.4)*			Reference
	Male	2001-2013	-2.3 (-4.0, -0.5)*					P=0.002
Age (years)	<20	2001-2013	3.5 (-1.0, 8.1)					P=0.81
	20 to 29	2001-2013	5.0 (2.3, 7.7)*					P=0.52
	30 to 39	2001-2006	-4.4 (-9.4, 0.9)	2006-2011	10.1 (2.5, 18.3)*	2011-2013	-7.0 (-25.4, 15.7)	P=0.03
	40 to 49	2001-2007	-2.2 (-5.8, 1.6)	2007-2013	8.8 (5.5, 12.2)*			Reference
	50 to 59	2001-2007	-2.2 (-5.4, 1.0)	2007-2013	6.0 (3.0, 9.1)*			P=0.01
	60 to 69	2001-2013	-1.5 (-3.2, 0.3)					P<0.001
	70+	2001-2013	-3.4 (-5.5, -1.1)*					P<0.001
Practice-level IMD quintile	Least deprived	2001-2007	-4.7 (-7.9, -1.4)*	2007-2011	8.6 (-1.1, 19.2)	2011-2013	-8.3 (-24.0, 10.6)	P=0.13
	Quintile 2	2001-2007	-3.7 (-7.2, -0.2)*	2007-2010	10.9 (-8.0, 33.7)	2010-2013	0.6 (-7.8, 9.8)	P=0.63
	Quintile 3	2001-2013	3.0 (0.6, 5.5)*					Reference
	Quintile 4	2001-2006	-6.7 (-11.3, -1.9)*	2006-2013	1.1 (-2.0, 4.4)			P=0.002
	Most deprived	2001-2013	1.4 (-0.5, 3.3)					P=0.64

[†] Trends shown are best fit to data for individual levels of each categorical variable. Test for parallelism P-value indicates strength of evidence for departure from parallel trend with reference level of categorical variable

* Evidence that Annual Percentage Change is greater than or less than zero at $\alpha=0.05$
