# Supplementary material for: Trends in the incidence of chronic fatigue syndrome and fibromyalgia in the UK, 2001–2013: a Clinical Practice Research Datalink study
Source: J R Soc Med. 2017 Mar 30;110(6):231–44. doi: 10.1177/0141076817702530 (PMC5499564; doi:10.1177/0141076817702530)
Supplement: Supplementary material [file JRS702530_supplementary_figures.pdf]

Supplementary Figure 1: Lewis plot of incidence rates in months 13-24 of follow-up

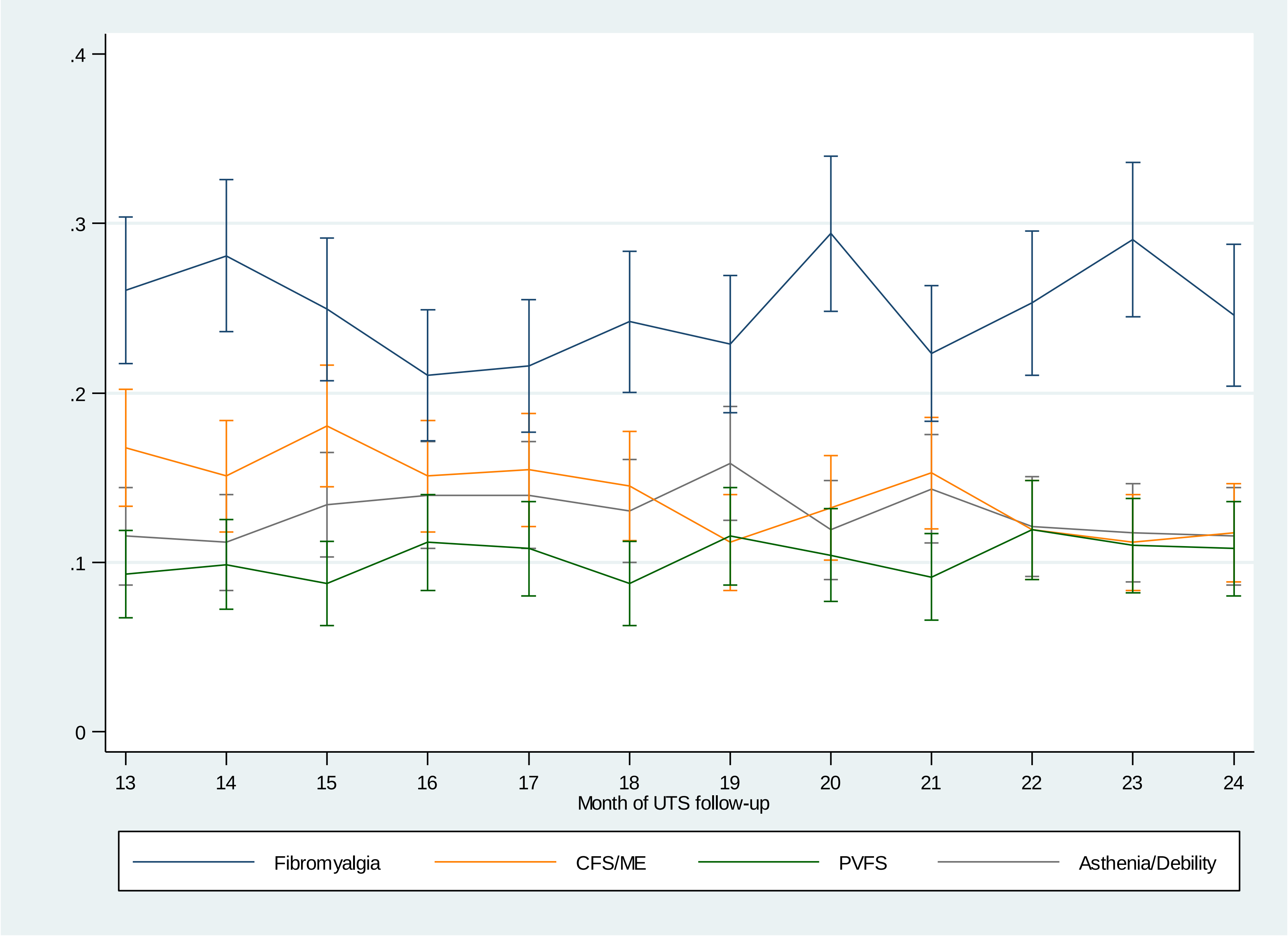

Supplementary Figure 2: Diagnoses of CFS/ME, fibromyalgia (FM), post viral fatigue syndrome (PVFS) and asthenia/debility

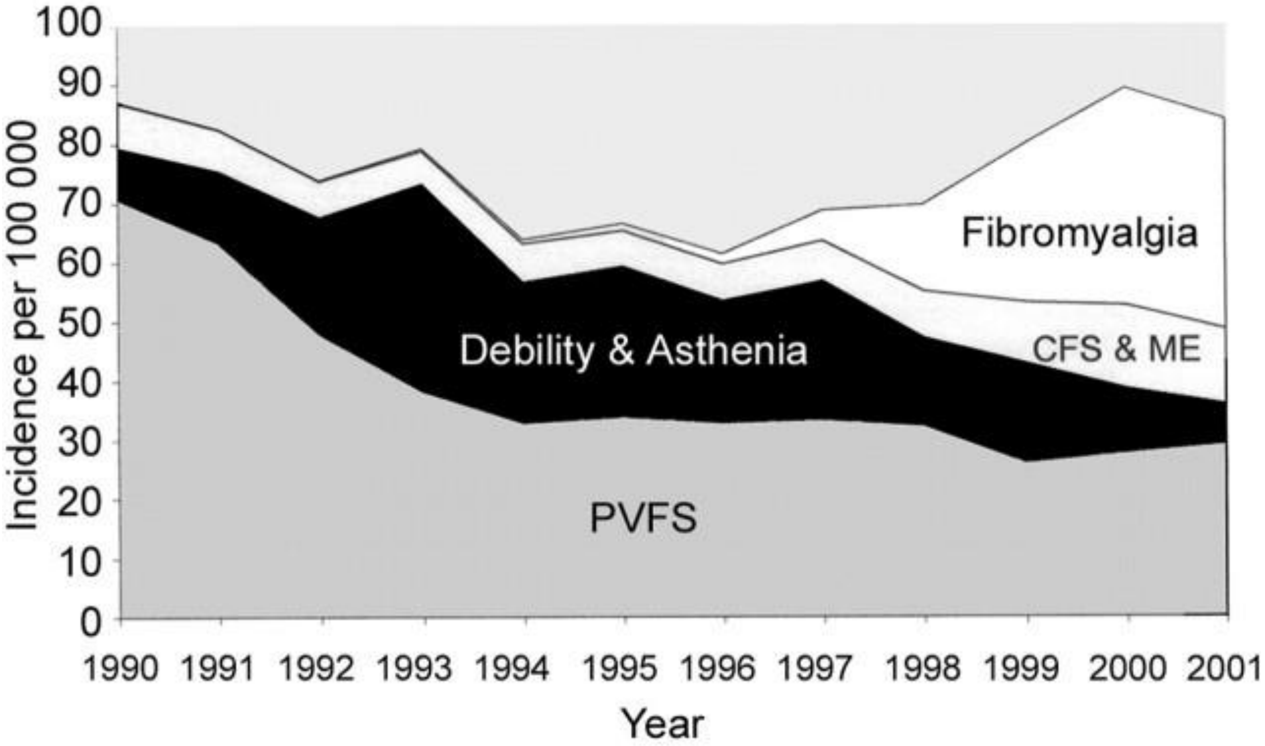

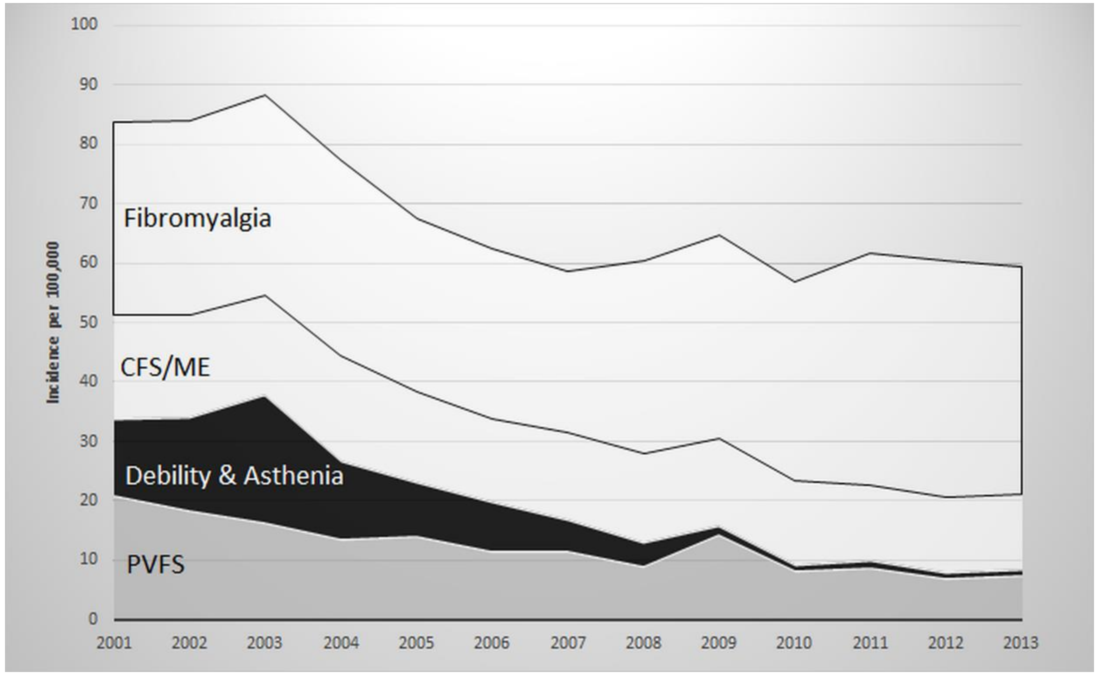

Gallagher AM, Thomas JM, Hamilton WT, White PD. Incidence of fatigue symptoms and diagnoses presenting in UK primary care from 1990 to 2001.
J R Soc Med. 2004;97(12):571-5 Copyright © 2004, The Royal Society of Medicine
